# An Open Science Peer Review Oath

**DOI:** 10.12688/f1000research.5686.2

**Published:** 2015-01-09

**Authors:** Jelena Aleksic, Adrian Alexa, Teresa K Attwood, Neil Chue Hong, Martin Dahlö, Robert Davey, Holger Dinkel, Konrad U Förstner, Ivo Grigorov, Jean-Karim Hériché, Leo Lahti, Dan MacLean, Michael L Markie, Jenny Molloy, Maria Victoria Schneider, Camille Scott, Richard Smith-Unna, Bruno Miguel Vieira, as part of the AllBio: Open Science & Reproducibility Best Practice Workshop

**Affiliations:** 1 Wellcome Trust – Medical Research Council Cambridge Stem Cell Institute, University of Cambridge, Cambridge, CB2 1QR, UK; 2 DNAdigest, Cambridge, UK; 3 University of Manchester, Manchester, UK; 4 Science for Life Laboratory, Uppsala University, Uppsala, Sweden; 5 The Genome Analysis Centre, Norwich Research Park, Norwich, NR4 7UH, UK; 6 European Molecular Biology Laboratory, Heidelberg, Germany; 7 Core Unit Systems Medicine, University of Würzburg, Würzburg, Germany; 8 DTU Aqua, Technical University of Denmark, Charlottenlund 2920, Denmark; 9 Software Sustainability Institute, Edinburgh, UK; 10 Open Knowledge Finland - Open Science Work Group, Helsinki, Finland; 11 The Sainsbury Laboratory, Norwich Research Park, Norwich, NR4 7UH, UK; 12 F1000Research, London, UK; 13 Department of Zoology, University of Oxford, Oxford, UK; 14 Michigan State University, East Lansing, MI, USA; 15 Department of Plant Sciences, University of Cambridge, Cambridge, UK; 16 School of Biological and Chemical Sciences, Queen Mary University of London, London, UK

## Abstract

One of the foundations of the scientific method is to be able to reproduce experiments and corroborate the results of research that has been done before. However, with the increasing complexities of new technologies and techniques, coupled with the specialisation of experiments, reproducing research findings has become a growing challenge. Clearly, scientific methods must be conveyed succinctly, and with clarity and rigour, in order for research to be reproducible. Here, we propose steps to help increase the transparency of the scientific method and the reproducibility of research results: specifically, we introduce a peer-review oath and accompanying manifesto. These have been designed to offer guidelines to enable reviewers (with the minimum friction or bias) to follow and apply open science principles, and support the ideas of transparency, reproducibility and ultimately greater societal impact. Introducing the oath and manifesto at the stage of peer review will help to check that the research being published includes everything that other researchers would need to successfully repeat the work. Peer review is the lynchpin of the publishing system: encouraging the community to consciously (and conscientiously) uphold these principles should help to improve published papers, increase confidence in the reproducibility of the work and, ultimately, provide strategic benefits to authors and their institutions.

## Open science and the future of peer-review

Science builds on itself. New knowledge is gained in the context of the enlightenment of earlier discoveries and, for the foundations to remain solid each discovery must be accurate and reliable. But science in the real world is messy, and advances haltingly and piecewise. Often, prior information is incomplete, so conclusions drawn need revising in the light of new, later evidence. This means that self-reference and self-checking are cornerstones of the scientific method; and, having reported our experiments, it is vital that readers are able to repeat them and understand how we reached our conclusions. Various studies have shown that, for a study to be successfully reproduced, information presented in the literature is often inadequate and the underlying data not readily available ^1^ - a significant drawback. Several commentators have concluded that weaknesses in the way that research investigations are currently conducted, and how their results are disseminated via article publication have become detrimental to the scientific process ^2– 7^. As the technological sophistication of science increases, and the equipment used becomes more specialised, the data generated are harder to represent in traditional media, and reporting how experiments were performed so that independent researchers can repeat them becomes progressively harder.

Open science is a movement that seeks to ensure that the results and the data of scientific research are, and continue to be, available to all. One way in which reproducibility issues can be tackled is through the use of open-science and open-data practices ^8, 9^. As attendees of the *AllBio: Open Science & Reproducibility Best Practice Workshop*, we discussed how the problem of keeping science transparent and reproducible in an increasingly technology-driven, and specialised, domain could be addressed.

One route, at the heart of scientific endeavour, is through the peer-review process. Peer review is an important gatekeeper and key component of scientific discourse. Before research findings can be formally accepted, they must be evaluated and commented upon by other domain experts, who provide advice about the quality or validity of the work to journal editors and/or readers (whether via pre-publication peer review, open peer review or post-publication peer-review systems). Importantly, peer review happens at a personal, rather than institutional, level and is carried out by individuals; it is therefore an ideal mechanism for engaging most researchers, given that all scientists peer review or are peer reviewed.

## Peer review oaths

The peer-review process is not infallible ^10– 12^; its weaknesses are many and varied, including the time available to perform thorough reviews, reviewers’ expertise, journals’ perception of relevance/interest/impact, and so on. Arguably, one of the most significant problems – certainly the one that generates most friction – is that reviewers can safely dispense self-serving and biased critiques, fully protected by the mask of anonymity. In some instances, anonymity may be the only pragmatic option, but in the interest of objectivity and constructive discourse it shouldn’t be the cornerstone of the peer-review process. Scientists have become sufficiently frustrated by these issues to devise *ad hoc* solutions to help safeguard the quality of reviews, and allow reviewers to affirm that they will review in an ethical and professional way, and encourage clearer review processes. This has led to the articulation of various reviewer’s oaths ( *e.g*., ^13– 15^); it is these that inspired our discussions and prompted this article.

An outcome of our debate was the formulation of an addition to these oaths that helps codify the role of reviewers, encouraging and promoting best practices to ensure that the science they review is as open and reproducible as possible. The amendment includes guidelines not only on how to review professionally, but also on how to support transparent, reproducible and responsible research, while optimising its societal impact and maximizing its visibility.

The open-science peer-review oath (which can be found on figshare for reuse ^16^) we therefore propose is as follows ( Box 1).

**Box 1. Open peer review oath.**

**Principle 1:** I will sign my name to my review

**Principle 2:** I will review with integrity

**Principle 3:** I will treat the review as a discourse with you; in particular, I will provide constructive criticism

**Principle 4:** I will be an ambassador for the practice of open science

## Towards ‘open science friendly’ reviews

### The declaration in your review

Peer-review oaths tend to be short declarations that reviewers make at the start of their written comments, typically dictating the terms by which they will conduct their reviews. The novelty in our amendment is the addition of a new declaration, which we recommend including in review preambles:

**I will be an ambassador for open science**

For it to be successful requires reviewers to follow the spirit of the statement throughout their reviews, which may involve a little additional work. The statement itself is derived from the rationale outlined below. It is for reviewers to decide how to implement this on a case-by-case basis – it *is* case specific, but nevertheless provides a framework for nurturing useful and hopefully fruitful discussions.

## Guidelines for open science reviewers

Here is how we envisage the review process from an open-science reviewer's point of view:

- I will work with you to help improve your research, as I believe that peer review should be an open, supportive and collaborative process. I will therefore sign my review and state my identity.
- I will, before I review, ensure that I have everything (raw data, detailed methods/protocols, *etc*.) necessary to make a complete, independent assessment.
- I will encourage the application, and, where necessary, provide guidance for any open-science best practices relevant to my field that would help support transparency, reproducibility, re-use and integrity of your research.
- I will check that any data and software code are consistent with the text, that any digital object identifiers and accession numbers are correct and correctly cited, and that any models presented are archived, referenced and accessible.
- I will check that the data and any software code and support documentation that underpin the published concept are made available in a manner that provides long-term unrestricted access.
- I will advise, where necessary, on how to achieve better transparency and availability (in terms of materials and methodology, data and code access, versioning, algorithms, software parameters and standards), with the understanding that adherence to best open-science practices may require further effort from you.
- I will support others in writing open reviews, where it is appropriate for me to do so.
- I will decline to review if I am not an appropriate reviewer (whether because of my expertise or because of my relationship with the author(s)). In doing so, I will provide journal editors with an honest appraisal of these issues, and will openly explain how I reached my decision so that alternative reviewers may be found.

## Conclusion

We believe that combining the open-science principle with some of the other key peer-review principles will help the scientific community to repeat published experiments, and to reach the same, or similar, conclusions.
