# Supplementary material for: An Open Science Peer Review Oath
Source: F1000Res. 2015 Jan 9;3:271. Originally published 2014 Nov 12. [Version 2] doi: 10.12688/f1000research.5686.2 (PMC4304228; doi:10.12688/f1000research.5686.2)
Supplement: Peer review history

# Peer review: Referee response for version 2

**DOI:** 10.5256/f1000research.6351.r7277

**Published:** 2015-01-22

**Authors:** Lawrence P Kane

**Affiliations:** 1 Department of Immunology, University of Pittsburgh, Pittsburgh, PA, USA

The authors have done a nice job responding to my critique of the original version, and the resulting revised version is much easier to read. Reading the other reviews, and the comments, I can see that this article is sparking a good amount of discussion on an important topic. Clearly this will not be the last word on the subject, and that is a good thing.

I have read this submission. I believe that I have an appropriate level of expertise to confirm that it is of an acceptable scientific standard.

# Peer review: Referee response for version 2

**DOI:** 10.5256/f1000research.6351.r7275

**Published:** 2015-01-20

**Authors:** Etienne Joly

**Affiliations:** 1 Equipe de Neuro-Immunogénétique Moléculaire (ENIGM), Bâtiment CNRS, IPBS CNRS Université Paul Sabatier, Toulouse, France

The manuscript has been improved compared to the first version. Despite the authors laudable intentions, I am still unconvinced that this will prove terribly useful to the scientific community, but I do not see any reason why this paper should not be indexed and referenced.

I have read this submission. I believe that I have an appropriate level of expertise to confirm that it is of an acceptable scientific standard.

# Peer review: Referee response for version 2

**DOI:** 10.5256/f1000research.6351.r7276

**Published:** 2015-01-12

**Authors:** Vitaly Citovsky

**Affiliations:** 1 Department of Biochemistry and Cell Biology, University of New York at Stony Brook, Stony Brook, NY, USA

The revised paper is much improved in terms of its (now appropriate and not tedious) length and clarity of statements. I still see a problem with the proposed absolute requirement (listed as the first principle) of disclosing the reviewer's identity. While noble in principle, I think it is unrealistic at this time; if so, compromising the first principle would appear to make the subsequent ones partly irrelevant. 

On the other hand, the authors clearly explained their decision to avoid addressing one specific issue of the reviewing process - frequent requests for tedious and time consuming experiments that do not contribute substantially to the main point of the paper, often precluding publication. As an experimental scientist, however, I do see this point as critical and think that its resolution would  contribute more to the fairness of review than disclosing the reviewer's identity.

Finally, I am still surprised that the related work by Bienz and Weston is not discussed and/or cited.

I have read this submission. I believe that I have an appropriate level of expertise to confirm that it is of an acceptable scientific standard, however I have significant reservations, as outlined above.

# Peer review: Referee response for version 1

**DOI:** 10.5256/f1000research.6078.r6800

**Published:** 2014-12-10

**Authors:** Lawrence P Kane

**Affiliations:** 1 Department of Immunology, University of Pittsburgh, Pittsburgh, PA, USA

The principles outlined here are important, and this piece is certainly timely. I have two suggestions to improve the manuscript.  First, as currently constituted, the manuscript is a bit repetitive, with a large text box recapitulating what is also laid out in much the same format in the body of the manuscript. Perhaps each of the major points could be expounded upon in the text. 

Second, while it is hard to argue with the specific tenets enumerated here, there are quite a lot of them. I guess my feeling is that for ideas like this to be more widely adopted that it would be helpful if the core principles could be boiled down to a more manageable size (which would be ideally presented in a text box). 

I applaud the authors for doing their part to increase openness in scientific publishing, something I agree is very much needed.

I have read this submission. I believe that I have an appropriate level of expertise to confirm that it is of an acceptable scientific standard, however I have significant reservations, as outlined above.

# Response

**Published:** 2015-01-10

**Authors:** Dan MacLean

**Affiliations:** The Sainsbury Laboratory, UK

Thanks for your helpful and candid report Larry. We agree the manuscript was a little repetitive and so we have made it much more succinct, by concentrating on the open science principle we want to champion. We have also condensed the oath into 4 principles with an accompanying rationale and made it available on FigShare, which should make it much easier to follow and reuse.

# Peer review: Referee response for version 1

**DOI:** 10.5256/f1000research.6078.r6987

**Published:** 2014-12-09

**Authors:** Suzanne Scarlata

**Affiliations:** 1 Department of Physiology and Biophysics, Stony Brook University, Stony Brook, NY, USA

I think this article makes many good points, and I also agree with the other critiques. However, I do see danger in full transparency. The problem is that significance of study can be  subjective and used in a biased way to sway readers towards a view of greater importance, in order to achieve a good standing with the authors.  At this point in time, it is important for editors of other non-transparent journals to remind reviewers of the key aspects of this oath – to critically read the paper and make constructive comments as best as they can.  Editors need to use their power to delete inappropriate reviews or reviewers.

I have read this submission. I believe that I have an appropriate level of expertise to confirm that it is of an acceptable scientific standard.

# Response

**Published:** 2015-01-10

**Authors:** Dan MacLean

**Affiliations:** The Sainsbury Laboratory, UK

Thanks Suzanne for your helpful and candid report. We have now satisfied the other reviews to make the oath clearer and simpler to use. We take your point that perceived impact can indeed sway readers and we certainly agree that as well as the scientific community practicing open science and reviewing openly,  journals and journal editors need to play a role in ensuring that reviewers are doing a job that ensures integrity - we believe they can use the principles of the oath to do this.

# Peer review: Referee response for version 1

**DOI:** 10.5256/f1000research.6078.r6802

**Published:** 2014-11-27

**Authors:** Etienne Joly

**Affiliations:** 1 Equipe de Neuro-Immunogénétique Moléculaire (ENIGM), Bâtiment CNRS, IPBS CNRS Université Paul Sabatier, Toulouse, France

This manuscript was written by participants of a workshop entitled "AllBio: Open Science & Reproducibility Best Practice Workshop" which took place at the TGAC in Norwich UK in September 2014. The alleged purpose of the oath and manifesto proposed in this manuscript is to make "transparency, reproducibility and citizen-scientist engagement the default parameters for performing sound research."

Those goals are clearly highly laudable, and I completely agree that the process of scientific refereeing would greatly improve by going 'open'. I do, however, feel very ill at ease about acting as a referee for this particular paper, for the following three main reasons:

1. This is not a scientific manuscript, and it neither contains data, nor reviews a scientific topic. I am thus left wondering why such a manuscript should need to be peer reviewed. After reading it, I certainly cannot  conclude that it is scientifically sound. The best I can conclude is that it is not scientifically unsound.
2. Although this manuscript is very short, I must say that I found it rather difficult to read because of its structure which contains many redundancies and of the fuzziness of its purpose. One aspect that particularly bothers me is the unquestioned assumption that open refereeing will improve reproducibility. The first thing the authors should clarify is what they mean by reproducibility since there are at least two types that I can think of:
  The first one concerns data reproducibility (or robustness). In other words will similar data, leading to the same conclusions, be obtained if the experiment is repeated (on a different day and/or with different samples and/or in a different place and/or by different people etc…). The second type of reproducibility relates to the capacity of other scientists to reproduce an experiment described in a published manuscript. I suspect that when they refer to 'science reproducibility', the authors refer to the latter type, although the first type is the most important one in my eyes.
3. The authors duly acknowledge that they have been inspired by reviewer's oaths previously proposed by others (refs 13-15), and if I am being completely honest, I do not find that the set of 17 rules they propose represent a significant improvement on those previously proposed oaths. Because I do not want to plagiarise Jonathan Eisen, I will simply suggest that the authors should very seriously consider following the many suggestions he has made on his blog to improve this paper ( http://icis.ucdavis.edu/?p=505 ), and especially the idea of reversing the Oath and the Manifesto.

I have read this submission. I believe that I have an appropriate level of expertise to confirm that it is of an acceptable scientific standard, however I have significant reservations, as outlined above.

# Response

**Published:** 2015-01-10

**Authors:** Dan MacLean

**Affiliations:** The Sainsbury Laboratory, UK

Thanks Etienne for your very helpful and candid report.  We agree that the article needed to be made clearer in order to convey its intentions to the reader and we have taken measures to do this by clearly elucidating the open science principle. We have also made it clear about what we mean by reproducibility, and how the robustness of research should mean that similar data should be able to produce the same conclusions. We have also simplified the oath on the advice given to us from you and the other reviewers to make it easier to read and reuse.

# Peer review: Referee response for version 1

**DOI:** 10.5256/f1000research.6078.r6797

**Published:** 2014-11-26

**Authors:** Chris Chambers

**Affiliations:** 1 School of Psychology, Cardiff University, Cardiff, UK

Overall, I believe this is a laudable proposal for a code of practice in academic peer review. In one sense it would be nice if such a code was unnecessary; after all, most of the practices outlined here should form part of any graduate training in science. In practice, of course, we know that the reality of peer review often falls short of achieving its aims. Here the authors outline 5 key principles and 17 key practices, which - if adhered to - would likely result in a more transparent and effective peer review mechanism.

While I am supportive of this initiative, I do have some concerns about the way it is presented and I also wonder how adhering to it could be incentivised. I will outline below some suggestions for possible improvement:

1. The authors begin by outlining the 17 key practices and only at the end do they group them together within governing principles. I think the overall structure of the paper would be clearer if this order were reversed - beginning with the five principles, explaining the key practices in each case that serve them, before returning to the code of practice or "oath" as the authors call it.
2. Some of the individual practices in the oath are somewhat ambiguous to me, and others seem to overlap. For instance, what exactly does it mean for a reviewer to "state their limits"? This is elaborated later under principle 2 but I would recommend clearer descriptions at all times. Other practices seem very similar, or least belong to common subsets of behaviour; e.g. practice 7 and practice 10; practice 2 and 6; practices 13, 14, 15 and 16 are all very similar and not clearly distinguished. With some careful attention I suspect the number of practices here could be halved, which would improve readability and likely uptake.
3. The language expanding on some of the core principles is perhaps little purple in places, e.g. " *I will use the majority of ‘doves’ to balance the ‘hawks’ in my review by sharing the content." *I'm not entirely sure what this means, but if it refers to the intention to balance sharp critical feedback with constructive suggestions or positive feedback, this could perhaps we said in a more straightforward way. The authors should bear in mind that not all readers will have English as a first language, and so more direct and less metaphorical phrasing could widen the appeal.
4. Two concerns with signed reviews need to be addressed. The first is the potential negative consequences felt by junior (non-tenured) scientists or minorities, who could, at least in theory, face severe repercussions for criticizing the work of senior/powerful colleagues who sit on editorial boards or grant panels. In my mind this conflict has never been properly addressed; while I believe it is completely reasonable for tenured scientists to be open and accountable in their reviews (I always sign mine), it is questionable whether this should be required uniformly across science. The second concern with open reviewing is the potential legal backlash of scientists being sued by litigious authors who feel aggrieved by a reviewer's published comments. We are seeing this already on PubPeer and elsewhere. This raises the question of who assumes legal responsibility for the content of a signed review. In the case of *F1000Research*, for instance, does it or will it provide legal indemnity to reviewers who choose to unmask themselves?
5. Finally, I would prompt the authors of this paper to consider, and ideally speculate on, ways their oath (or code of practice as I would call it) might be implemented and incentivised in science. Could it, for instance, be worked into the next REF in some way? How could this be achieved and what challenges would need to be overcome? How does this initiative relate to other emerging group-led initiatives, such as the Agenda for Open Research (https://agendaforopenresearch.org/)? There authors have no shortage of good intentions but as we know, there lies a world of groupthink and social inertia between good intentions and good practices.

Regardless of the above concerns, I applaud this much-needed call for greater transparency in the peer review process.

I have read this submission. I believe that I have an appropriate level of expertise to confirm that it is of an acceptable scientific standard, however I have significant reservations, as outlined above.

# Response

**Published:** 2015-01-09

**Authors:** Michael Markie

**Affiliations:** F1000Research, UK

Thanks Chris for your very helpful and candid report. Like all of the reviewers have suggested we have simplified the oath and made it much clearer and simpler to use. Yes, we agree that some of the language used was difficult for non-native English speakers and hence we have made it clearer to read in places that may of caused confusion. We also agree with your point about new/junior reviewers who may feel exposed by openly reviewing someone more senior than them and have acknowledged that in the guidelines. Here at *F1000Research, *we have implemented a system of co-authoring referee reports to help support junior researchers who feel that on their own they may be subject to repercussions from the authors. With regards to the legality of an open review on *F1000Research*, reviewers submit their report according to our terms and conditions which clearly state the report will be published under a CC BY license, and that the report must not be defamatory. However as a publisher we are also conscious about each review that is published on the site and we have system in place to ensure that nothing libel would be intentionally published. More generally, openness and transparency in signing a report means that they are on the whole much more civil and are of a constructive nature that encourages dialogue. We believe the peer review process should be a collaborative process and hence that is the environment we try to provide. Here is a letter of response from Rebecca Lawrence, Managing Director of *F1000Research*, recently published in response to the recent libel story from the anonymous review on PubPeer: http://www.timeshighereducation.co.uk/comment/letters/an-anonymity-problem/2017051.article. Finally, just after we published the review we became aware of the Agenda for Open Research who have similar goals that align with us and they were kind enough to link to the oath in their guideline for reviewers. We fully support their initiative, and we will seek opportunities to collaborate where possible. You are right, it would be great to push to get open reviewing to be recognised as an official part of the REF (and other funding bodies) and acknowledged as a measurable scientific output. Here at *F1000Research* we are taking steps to make this happen; currently we mint each report with a DOI and we are heavily involved in the Peer Review Service projec t in collaboration with ORCID and CASRAI.

# Peer review: Referee response for version 1

**DOI:** 10.5256/f1000research.6078.r6804

**Published:** 2014-11-25

**Authors:** Vitaly Citovsky

**Affiliations:** 1 Department of Biochemistry and Cell Biology, University of New York at Stony Brook, Stony Brook, NY, USA

This article addresses a very important issue of peer review. Although, many of us tend to regard it in a way of "if it ain't broke, don't fix it", we also are often frustrated with the process and wish it were more fair, transparent and to the point (rather than requesting numerous experiments that are only tangential to the paper's main message). Thus, constructive approach to potential improvement of the peer review system is always welcome and should be encouraged. With this in mind, I support, in principle, publication of this manuscript. On the other hand, the way that the authors present their ideas is not perfect. Here is why:

First they make a huge emphasis in their introductory remarks on experimental reproducibility and societal impact. However, their "oath" does not address these issues specifically and constructively; instead, it offers declarative statements which are mostly trivial and already represent part and parcel of today's peer review process (e.g., "I will comment on how well you have achieved transparency, in terms of materials and methodology, data and code access, versioning, algorithms, software parameters and standards, such that your experiments can be repeated independently"). 

Second, the authors do not address clearly one very important aspect of potential improvement of the review process which is "To request further experiments only as a last resort, and only if they are essential to validate the conclusions of the paper. No experiments extending the study beyond its conclusions, or with unreasonable cost or time implications, should be proposed. An estimate of time required for the additional work should be provided." This is a quote from Mariann Bienz and Kathy Weston who also addressed the issue of peer reviewing ( http://elifesciences.org/elife-news/a-reviewers-charter).

Third, the "oath" is unreasonably long. Again, I suggest to look at the post by Bienz and Weston, which unlike the present paper, is much clearer, laconic and to the point.

Fourth, the authors quite nonchalantly suggest to overturn the cornerstone of the present review system - its anonymity. What is the chance for that? The reason for such a step should be well reasoned and substantiated.

I have read this submission. I believe that I have an appropriate level of expertise to confirm that it is of an acceptable scientific standard, however I have significant reservations, as outlined above.

# Response

**Published:** 2015-01-10

**Authors:** Dan MacLean

**Affiliations:** The Sainsbury Laboratory, UK

Thanks Vitaly for your very helpful and candid report. We have now explained what we hope to achieve from the oath, how we think it could address the issue of reproducibility and how the oath could have a positive societal impact in the scientific community. We believe at the researcher level there is a unique opportunity to help spread open science practices when reviewing articles and help improve what the community as a whole believes should be available in a paper in order for it to be reproduced. We agree with your point about not asking for additional experiments, however we have decided to concentrate fully on elucidating the requirements of the open science principle specifically and not the other principles. As with the other reviewers and comments we have taken all of your advice and shortened the oath to make it simpler for reuse. We also agree with your point about anonymity and we have discussed this in the manuscript on why we should move towards open peer review.
